# Transcriptional changes measured in rice roots after exposure to arsenite-contaminated sediments

**DOI:** 10.1007/s11356-017-0515-z

**Published:** 2017-11-13

**Authors:** Alexandra Brinke, Georg Reifferscheid, Roland Klein, Ute Feiler, Sebastian Buchinger

**Affiliations:** 1 German Federal Institute of Hydrology, Am Mainzer Tor 1, 56068 Koblenz, Germany; 2 Department VI, Trier University, Biogeography, 54286 Trier, Germany

**Keywords:** Aquatic plants, Freshwater toxicology, Biomarkers, Ecotoxicogenomics, Sediment toxicity

## Abstract

Transcriptional analyses are discussed to provide a deeper understanding of the molecular mechanisms underlying toxic effects. Thus, they can complement classic ecotoxicological test methods and potentially allow the identification of biomarkers associated to the exposure of chemical stressors and or adverse biological effects. This feasibility study intended to identify a set of potential gene expression biomarkers for arsenite-exposure in rice roots that could complement the informative value of an existing sediment-contact test with rice. A sediment-contact test with *Oryza sativa* with the parameters inhibition of root and shoot elongation as phenotypic endpoints was used as basis. Rice plants were exposed to arsenite-spiked sediments. Transcriptomic changes in response to arsenite were observed by means of cDNA-microarray analysis regarding the whole-transcriptome at two sublethal arsenite concentrations. In order to identify candidate biomarker genes, differentially expressed genes were identified. Arsenite-induced differentially expressed genes were significantly associated with gene ontology (GO)-terms that indicated a general stress response. Of the differentially expressed genes, five genes were selected and their expression was measured at seven arsenite concentrations by means of qPCR in order to obtain their expression profiles. Three candidate biomarker genes showed a dose-dependent upregulation, while two showed no clear dose-dependent expression. The expression of all candidate biomarkers was also assessed in rice plants grown on two arsenic-contaminated natural sediments, but only one biomarker gene showed the expected upregulation.

**Electronic supplementary material:**

The online version of this article (10.1007/s11356-017-0515-z) contains supplementary material, which is available to authorized users.

## Introduction

In sediment quality assessment, the chemical analysis of extracts from sediments reflects—under the assumption of a quantitative extraction method—the total contamination level of the sediment. This approach neglects the aspect of the bioavailability of sediment contaminants. Thus, the measured total contamination might overestimate the actual hazard. Furthermore, effects caused by the measured substances or mixtures of substances remain unknown. Therefore, bioassays—especially sediment-contact bioassays—are highly valuable complementary tools to chemical analysis as they capture only bioavailable fractions of contaminants (Traunspurger et al. 1997). In such whole-organism bioassays, adverse effects are often assessed as phenotypic changes. However, the first step in a chain leading to adverse effects on the phenotype is in most cases stressor-induced alterations on the transcriptomic level (Nuwaysir et al. 1999). This means alterations on the transcriptomic level are measurable prior to effects on the phenotypic endpoints of common bioassays. In addition, weak or chronic effects can be measurable as transcriptional changes, also if these effects will not become manifest as adverse effects on the phenotype in an acute test and can thus stay undetected (Nuwaysir et al. 1999). Effects on the transcriptome can be assessed as the induction of stress-related pathways, transcriptomic patterns, or single biomarker genes. However, to reliably identify stress on the transcriptomic level, especially if genes with unknown function are involved, transcriptional changes have to be clearly linked to distinct stress-responses on the phenotype. This idea was proposed by Paules (2003) as the concept of “phenotypic anchoring”. Studies combining aquatic whole-organism bioassays with transcriptomic endpoints were already published for different test species such as *Caenorhabditis elegans* (Menzel et al. 2009), *Danio rerio* (Hausen et al. 2017; Kosmehl et al. 2012), *Daphnia magna* (Heckmann et al. 2008), *Melitta plumulosa* (Hook et al. 2014a, b, c), or *Elliptio complanata* (Robertson et al. 2017).

Despite their essential role in aquatic ecosystems, higher plants as test organisms are generally less represented in ecotoxicological bioassays, especially in sediment toxicity assessment. One aquatic bioassay with *Lemna minor* (ISO 2005) and one sediment-contact bioassay with *Myriophyllum aquaticum* (ISO 2013) are standardized, however still rarely used in sediment assessment. Therefore, studies combining aquatic bioassays with higher plants with transcriptomic analysis are underrepresented. Regier et al. (2013) identified subsets of differentially expressed genes in *Elodea nutallii* that were specifically induce by mercury or cadmium and that are hypothesized to work as biomarker for heavy metal exposure in fresh water or sediments. However, similar approaches with higher plants, especially monocots, are still scarce, especially using sediment-contact tests.

The present study presents the first step of a feasibility study, challenging the question if a sediment-contact test using *Oryza sativa* might be complemented by means of candidate biomarker genes (CBGs) as transcriptional endpoints for sediment-contamination. Against this background, transcriptional changes in roots of *Oryza sativa* after exposure to arsenite-spiked artificial sediments were measured in order to identify transcripts with strong arsenite-induced changes of abundance that can be further characterized as CBGs for arsenite exposure. The selection of root tissue for the analyses of biomarker expression is based on the following rationale: the root is in direct contact to the sediment to be investigated and the organ responsible for uptake and possibly metabolization of sediment-associated contaminants (Wang et al. 2015). After the initial uptake, compounds are distributed in the whole plant. Thus, a concentration gradient from root to shoot might to be expected. In fact, in the case of arsenic, it is known that its concentrations in rice decreases from roots over shoots to seeds (Souri et al. 2017). Therefore, it was concluded that the root tissue is likely to be the first organ affected by toxic effects leading to the decision that biomarker expression was measured in rice root tissue.

In the course of the presented study, the following hypotheses were experimentally challenged: (I) Treatment and references, obtained from exposures of rice roots to arsenite-spiked artificial sediments, show significant differences in gene expression. (II) Arsenite-induced differentially expressed genes (DEGs) can be identified from the total gene set on the microarray. (III) CBGs show a dose-dependent expression in response to arsenite-induced stress.

## Material and methods

### Exposure of rice plants

*Oryza sativa ssp. indica* was used as test organism. If not stated otherwise, the sediment-contact bioassay using *O. sativa* was performed as published elsewhere (Brinke et al. 2015).

For the assessment of the transcriptional changes by means of DNA-microarray analysis, pre-germinated rice seeds were exposed for 7 days to arsenite-spiked artificial sediments at concentrations of 11 mg kg^−1^_dw_ or 15 mg kg^−1^_dw_ arsenite [As(III); CAS 7784-46-5; Aldrich] referred to as As_low_ and As_high_. These concentrations are above and below the EC_50_ for root elongation at 13 mg kg^−1^_dw_ added arsenite which was determined by Brinke et al. (2015). Root samples were taken from three independent exposures. Per exposure, four replicates per concentration were used for the measurement of the phenotypic endpoint root and shoot elongation. Four additional replicates per concentration were characterized by DNA-microarray analyses. Thereof, a total sample size of *n* = 12 root samples per arsenite concentration were hybridized in the microarray experiments. As each exposure was accompanied by reference samples, a total sample size of *n* = 36 reference samples were hybridized in the microarray experiments.

The expression profiles of single candidate biomarker genes were measured by means of quantitative PCR (qPCR). Therefore, rice seedlings were exposed for 7 days to arsenite-spiked artificial sediments at concentrations of 5, 7, 9, 11, 13, and 15 mg kg^−1^_dw_ added arsenite_,_ as well as to two arsenic-contaminated natural sediments sampled from the German Nahe river with the two sampling sites Niedernhausen, harbor (NH-N), and Bad Münster am Stein, harbor (BMAS-N). The concentrations of arsenic were NH-*N* = 13.3 mg kg^−1^_dw_ and BMAS-*N* = 15.6 mg kg^−1^_dw_. Sediment characteristics including heavy metal concentrations are given in Table S1. Root samples were taken from three independent exposures. Per exposure and concentration, four technical replicates were used for the measurement of the phenotypic endpoint root and shoot elongation; three further technical replicates per concentration and exposure were analyzed for gene expression changes by means of qPCR.

Roots used for the transcriptional analyses, either by microarray or qPCR, were treated as follows: The root tissue was rinsed with distilled water and then immediately flash frozen in liquid nitrogen. Two to three plants, depending on the amount of root tissue (about 100 mg in total), were pooled as replicate in a 2-ml reaction tube. For long-term storage, the roots were kept at − 80 °C.

### RNA extraction and QC measures

The RNA from shock frozen root tissue was extracted using the RNeasy Plant Mini Kit (QIAGEN) according to the manufacturer’s protocol. To remove potential DNA contamination, the RNA samples were treated with DNase (peqGOLD DNase I Digest Kit by Peqlab Biotechnologie GmbH) according to supplier information. The integrity and amount of the isolated nucleic acid was checked by means of the RNA 6000 Nano Kit (Agilent) on the Agilent Bioanalyzer according to the manufacturer’s protocol (Table S2). Only samples with RNA integrity numbers (RIN) above 8 were used for microarray analysis or qPCR. The purity of samples was assessed by the 260/280 nm-ratio using the IMPLEN NanoPhotometer P330. Only samples with 260/280 nm ratios between 1.8 and 2.0 were selected for subsequent microarray analysis or qPCR.

### DNA-microarray analysis

The DNA-microarray analysis was performed using 0.2 μg high quality total cRNA generated with the Low Input Quick Amp Labeling Kit, One-Color according to the manufactures protocol. The sample preparation and the hybridization were performed according to Agilent’s protocols for one-color microarray-based gene expression analysis. The scanning of the chips was performed on the Agilent Technologies Scanner G2505C according to the manufacturer’s protocol. Expression data were extracted with the Agilent Feature Extraction software. Raw data were uploaded in the ArrayExpress Archive of Functional Genomics Database (Experiment ArrayExpress accession: E-MTAB-5430) (Parkinson et al. 2011). For group-comparisons, raw data of the respective samples were quantile normalized. Quantile normalized data were processed as log_2_-fold change (LFC). All following statistical analyses were performed with the TIGR MultiExperiment Viewer (MeV) software (Saeed et al. 2003). DEGs were identified by means of the three statistical methods: linear models for microarray data (LIMMA), (two-class unpaired) significance analysis of microarrays (SAM), and *T* tests. Genes were only selected as DEGs if they were identified as differentially expressed by all statistical methods. This approach resulted in the rejection of false-positive genes, which were selected as significant due to outlier values of single replicates. The cutoff for the SAM was set to a value of 50 ± 3 genes that were included in the further analysis. The calculated false discovery rate was always 0%. The *T* test was calculated using the Welch approximation with *p* values based on a *t* distribution and an overall threshold value of *α* = 0.001. The significance was determined by means of the adjusted Bonferroni correction. From the identified DEGs, genes were selected as CBGs if they showed high relative expression changes while ensuring a low variance between the replicates. Expression differences of As_low_ or As_high_ versus the references were visualized in principal component scatterplots (PCA) with the centering mode based on the median. To gain a better insight into arenite’s mechanism of action in rice plants, the DEGs for As_low_ and As_high_ were associated with GO-terms for biological functions using the rice array platform (Cao et al. 2012). Prior to this, the genes had to be transformed from the RAP ID to the corresponding pub_locus IDs using the Rice Annotation Project Database’s ID converter (Sakai et al. 2013). Only GO-terms with hyper *p* values below *p* = 0.01 were regarded as significantly induced.

### qPCR

To obtain dose-response curves of the candidate biomarker genes, their expression was analyzed by means of qPCR over a range of seven arsenite concentrations as described above. First strand cDNA was prepared from DNase-treated total RNA in a total volume of 20 μl with the Affinity Script qPCR cDNA Synthesis Kit according to the manufacturer’s protocol. Two microliters of the DNase-treated RNA suspension were used for cDNA Synthesis. RNA amounts for cDNA synthesis ranged from 3 to 31 ng of RNA according to recommendations in the manufacturer’s protocol (3 pg–3 μg total RNA). The qPCR analyses were carried out using the MX3005P device (Stratagene, La Jolla, CA, USA). The reactions were prepared according to the manufacturer’s protocol for Brilliant III SYBR Green QPCR Master Mix (Stratagene) containing 2 μl prediluted cDNA in a 25 μl reaction. Information on primer sequences, as well as concentrations and temperature profiles used for the qPCR are given in Tables S3 and S4. The amplification was followed by a melting curve analysis to confirm PCR product specificity. During the optimization of the gene specific qPCR protocols, product specificity and absence of primer dimers were further confirmed using the Agilent DNA 1000 Kit run on Bioanalyzer (Agilent). The experimental threshold (Cq) was calculated using the algorithm enhancements provided by the MxPro Mx3005P v3.00 software. The baseline was adjusted manually to the lag phase of the curve. All samples were run in technical triplicates and the mean value of each triplicate was used for further calculations. PCR efficiencies were calculated based on the kinetics of individual PCR reactions for each replicate. The expression changes given as relative expression ratio (RER) were determined as efficiency corrected calculation models based on multiple samples.(Pfaffl 2004) The “eukaryotic elongation factor 1 alpha” (eEF1a) was used for data normalization. Primer sequences for eEF1a were obtained from (Jain et al. 2006). Correlations between the RER for each gene and the inhibition of root and shoot elongation for rice plants grown on artificial arsenite-spiked sediment were conducted by means of spearman rank correlation. The results of this analysis are given as correlation matrix (Table S5). The dose-response curves for the five candidate biomarker were fitted using a 3-parametric sigmoidal curve fitting. The statistical analyses of qPCR data were conducted by means of Systat Software’s SigmaPlot 12.3.

### Data availability

Supporting data of the microarray analyses are available through the Array Express Archive of Functional Genomics Database (Experiment ArrayExpress accession: E-MTAB-5430). Further data, associated metadata, and calculation tools are available from the corresponding author.

## Results and discussion

The present study attempted to complement a sediment-contact test using *Oryza sativa* with the identification of CBGs. Therefore, the transcriptome of rice roots was measured by DNA-microarray analysis after exposure to two concentrations of arsenite (As_low_ and As_high_) in parallel to a characterization of the root elongation (Hypothesis I). Based on the DNA-microarray data arsenite-dependent DEGs were identified at both arsenite concentrations (Hypothesis II). For an elucidation of arenite’s mode of action in rice plants, DEGs were associated to GO-terms for biological functions. Of the DEGs, the most robust DEGs were selected as CBGs and characterized in more detail by means of qPCR over a broader range of arsenite concentrations (Hypothesis III).

### Phenotypic changes induced by arsenite-spiked artificial sediments.

After exposure of the rice plants to arsenite-spiked artificial sediments, roots and shoots showed a clear dose-dependent inhibition of elongation. The concentration-response curve for the inhibition of root and shoot elongation is shown in Fig. S1. The EC_50_ for the inhibition of root and shoot elongation on arsenite-spiked sediments was calculated as 13 and 20 mg kg^−1^_dw_, respectively, based on the combined results obtained from this and the results from a previously published study (Brinke et al. 2015). The inhibition of root and shoot elongation were found to be correlated (*r*_s_ = 0.943; *p* = 0.0167).

On artificial sediments, root elongation was the more sensitive endpoint for arsenite-induced stress compared to shoot elongation.

### Identification of DEGs from the whole gene set

**Fig. 1:**
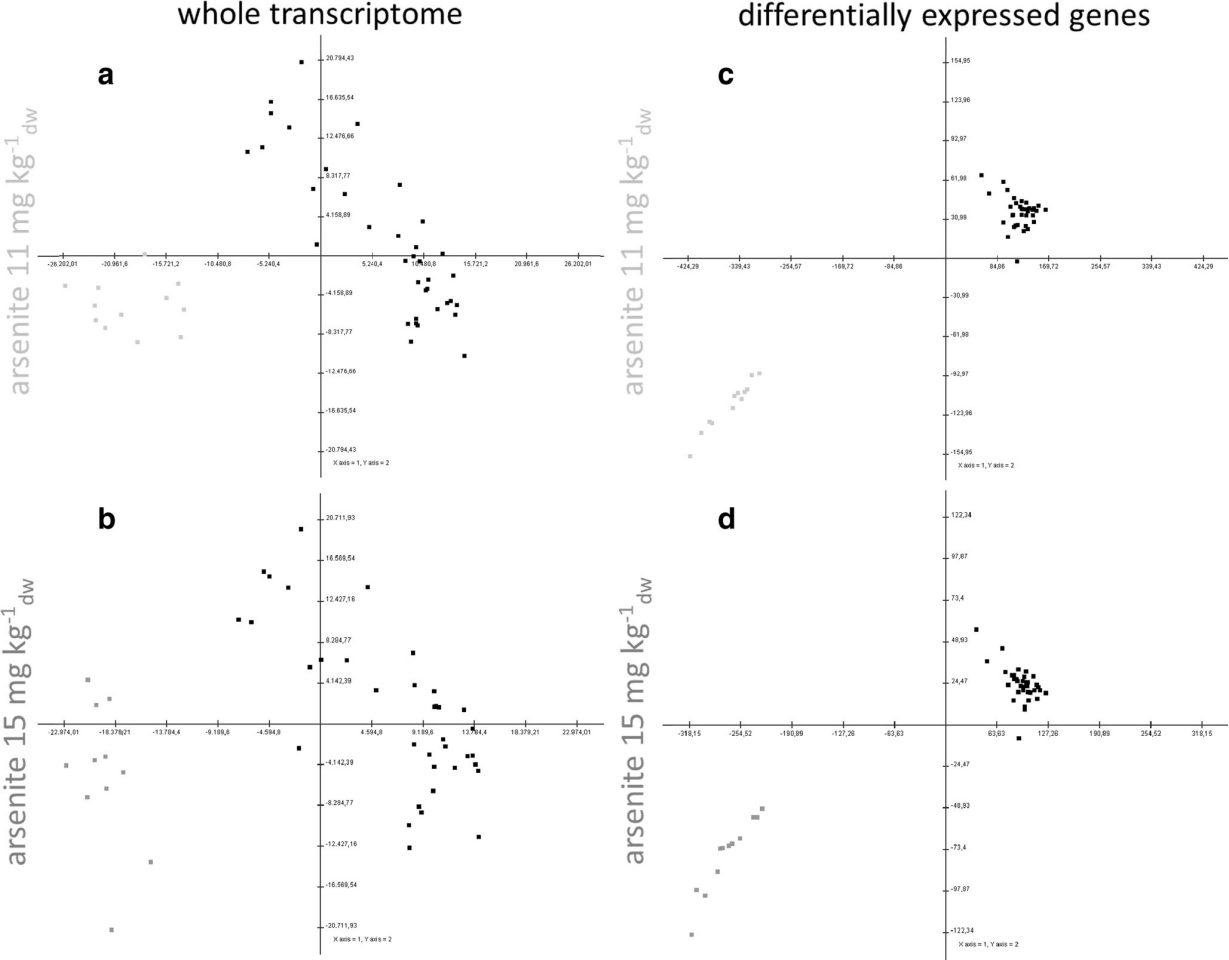
The impact of 11 mg kg^−1^_dw_ arsenite (**a**, **c**) and 15 mg kg^−1^_dw_ arsenite (**b**, **d**) on the whole transcriptome (**a**, **b**) as well as on differentially expressed genes (**c**, **d**) are displayed in a matrix of principal component scatterplots. Treatments and references are represented as dots, whereas the treatments exposed at 11 mg kg^−1^_dw_ are light gray dots (*n* = 12), the treatments exposed at 15 mg kg^−1^_dw_ are dark gray dots (*n* = 12) and the references are black dots (*n* = 36). PCA was performed using LFC values (treatment/median reference)

The impact of arsenite on the whole transcriptome in root tissue is visualized by a principal component analyses (PCA) shown in Fig. 1a. A clear separation between the treatment and reference samples was achieved at both arsenite concentrations (for eigenvalues see Table S7). Expected arsenite-induced effects on the transcriptome were thus proven.

To identify DEGs from the whole gene set, three different statistical analyses were performed as described in “Material and methods”. In total 17,145 genes (SAM 53 genes, LIMMA 17066 genes, *T* test 4164 genes) for As_low_ and 17,487 genes (SAM 53 genes, LIMMA 16724 genes, *T* test 3672 genes) for As_high_ were identified as significantly expressed by at least one of three applied statistical methods. For a robust identification of DEGs that serve as a pool for the final selection of CBGs, the results of three different statistical analyses for the DEG detection were combined. This was necessary, as the probability of false-positive results should be minimized as far as possible. However, despite the high number of references included in the analyses, by applying only one statistical standard method too many genes show false-positive results and were mistakenly identified as significant. False-positive results might occur in cases when only one sample shows high expression changes. The highest probability for false positives was observed in case of the *T* test. For both concentrations 53 DEGs each were identified, of which 46 genes were upregulated in As_low_ and 50 DEGs were upregulated in As_high_. As As_low_ and As_high_ had 46 DEGs in common, a high similarity was assumed and proven as no significant expression changes were found between As_low_ and As_high_, analyzed by means of a *T* test including the DEGs for each concentration. The LFCs of the selected DEGs are given in Table S6 of the Supporting Information. To assess the quality of this approach for the selection of DEGs, a PCA was performed based on the identified DEGs (Fig. 1b). For both arsenite concentrations, the eigenvectors changed from around 28% explainable variance captured by the whole gene set to more than 85% explainable variance by the DEGs only (see Table S7).

### Functional annotation of DEGs

In order to better interpret the observed arsenite-induced effects on the molecular and macroscopic level, DEGs were annotated with putative functions by means of a GO-term enrichment (Table 1). The analysis implies a high degree of similarity with regard to biological functions affected by As_low_ and As_high_ treatment (Table 1). GO-terms that were significantly associated only with As_high_ are “glutathione biosynthesis,” “oxidation reduction,” and the “L-arabinose metabolic process.” The suggested GO-term with the highest significance under As_low_ was “response to stress” which was also a prominent but less significant GO-term under As_high_ exposure. With increasing arsenite concentration, the significance of the term “response to stress” is superseded by the detoxification mechanism “glutathione biosynthesis”, which is linked to the term “oxidation reduction”. Verbruggen et al. (2009) reviewed the increasing GSH-synthesis under metal or metalloid stress as “a means of increasing metal(loid) binding capacity as well as a way to increase cellular defense against oxidative stress.” “Glutathione synthesis” is induced by heavy metal stress, as it acts as substrate for the formation of phytochelatins that play an important role in the detoxification of several metals and metalloids (Verbruggen et al. 2009). The related detoxification of arsenite in plants was described as the formation of glutathione-As(III) complexes, which can either be transported into the vacuole by an unidentified ABC transporter, or act as substrate for phytochelatin synthase resulting in complexation (Briat 2010; Schmöger et al. 2000; Verbruggen et al. 2009). In the presented study, the GO-term “glutathione biosynthesis” was suggested based on the upregulation of the related transcripts for glutamate-cysteine ligase and glutathione-S-transferases (GST). In accordance to the presented findings, Chakrabarty et al. (2009) described a dose-dependent glutathione reductase level in roots of rice seedling induced by arsenite. Ahsan et al. (2008) detected a dose-dependent upregulation of total glutathione and glutathione reductase in rice roots under arsenate stress in a proteomic study. They also found an arsenate-induced upregulation of S-adenosyl-L-methionine (SAM), a biological methyl donor taking part in several transmethylation reactions in plants. A SAM-dependent carboxyl methyltransferase was also found to be upregulated in the present study under arsenite stress at both concentrations. However, these changes were not significant. Further, identified GO-terms are discussed in the context of the detailed characterization of CBGs by qPCR (see section “Expression profiles of the CBGs”).

**Table 1:** GO-terms for biological functions that were significantly associated with the DEGs for As_low_ and/or As_high_

GO ID	GO name	Hyper *p* value		Gene model/	Description (RAP-DB)
		Aslow	Ashigh	Symbol of CBG	
GO:0006750	Glutathione biosynthetic process	N/A	0.0000	Os05g0129000	Glutamate—cysteine ligase, chloroplast precursor, putative, expressed
				Os12g0263000	Glutathione synthetase, chloroplast precursor, putative, expressed
GO:0006950	Response to stress	0.0000	0.0008	Os02g0527300	HSF-type DNA-binding domain containing protein, expressed
				Os02g0758000	Heat shock 22 kDa protein, mitochondrial precursor, putative, expressed
				Os03g0266900	Hsp20/alpha crystallin family protein, putative
				HSFA2A	HSF-type DNA-binding domain containing protein, expressed
GO:0008152	Metabolic process	0.0023	0.0014	Os01g0692000	Glutathione S-transferase, putative, expressed
				Os03g0154000	Expressed protein
				Os03g0283100	IN2-1 protein, putative, expressed
				HSFA2A	HSF-type DNA-binding domain containing protein, expressed
				Os03g0757600	UDP-glucoronosyl and UDP-glucosyl transferase domain containing protein, expressed
				UDPGT	anthocyanidin 5,3-O-glucosyltransferase, putative, expressed
				Os10g0527400	glutathione S-transferase GSTU6, putative, expressed
				Os10g0568900	haloacid dehalogenase-like hydrolase family protein, putative, expressed
GO:0009409	Response to cold	0.0049	0.0045	OsABI5	Similar to ABA response element binding factor (Fragment).
GO:0009414	Response to water deprivation	0.0049	0.0045	OsABI5	Similar to ABA response element binding factor (Fragment).
GO:0009651	Response to salt stress	0.0059	0.0054	OsABI5	Similar to ABA response element binding factor (Fragment).
GO:0009737	Response to abscisic acid stimulus	0.0078	0.0072	OsABI5	Similar to ABA response element binding factor (Fragment).
GO:0010152	Pollen maturation	0.0030	0.0027	OsABI5	Similar to ABA response element binding factor (Fragment).
GO:0046373	L-arabinose metabolic process	N/A	0.0072	Os01g0627800	Alpha-N-arabinofuranosidase A, putative, expressed
GO:0055114	Oxidation reduction	N/A	0.0068	Os03g0320100	cytochrome P450 72A1, putative, expressed
				Os04g0339400	oxidoreductase, aldo/keto reductase family protein, putative, expressed
				Os07g0418500	cytochrome P450 72A1, putative, expressed

*N/A* not available

### Expression profiles of the CBGs

**Fig. 2:**
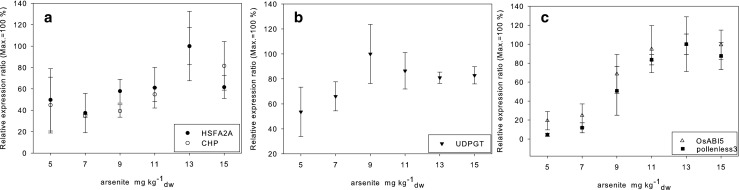
RER of the candidate biomarkers at five arsenite concentrations measured by means of qPCR (*n* = 3). For better comparability between the expression profiles, the RER are normalized to 100%

After having identified arsenite-induced changes on the transcriptome of rice roots, the next step in the present study was the identification of suitable gene expression biomarkers for the assessment of sediment-associated arsenite contamination. Only those genes were considered as CBGs that showed the strongest expression changes while the means LFCs showed low variances in the DNA-microarray analyses. To identify CBGs for arsenite exposure as further endpoints in the sediment-contact bioassay, we hypothesized that the selected CBGs should show a continuous dose-dependent expression in response to arsenite-induced stress. In the current study, a subset of five CBGs was chosen out of all arsenite-induced DEGs (Table S8). These were four transcripts with known functions: HSFA2A (HSF-type DNA-binding domain containing protein, expressed.), UDPGT (UDP-glucuronosyl/UDP-glucosyltransferase family protein), OsABI5 (Similar to ABA response element binding factor (Fragment).), and pollenless3 (Similar to pollenless3.), as well as CHP (conserved hypothetical protein.), a transcript with unknown function. The expression levels of the selected CBGs were measured by qPCR after exposing the rice to artificial sediment samples spiked with seven different concentrations of arsenite. The resulting dose-response relationships are shown in terms of normalized RERs in Fig. 2 and Table S9 (not normalized).

### Expression profiles and biological functions of HSFA2A and CHP

HSFA2A (Os03t0745000–01) and the CHP (Os05t0296800–02) showed a strong increase of expression already at the lowest test concentration of 5 mg kg^−1^_dw_ (Fig. 2a). However, the expression levels did not increase substantially with higher arsenite concentrations. The HSFA2A transcript is related to heat shock protein (HSP) synthesis (Nishizawa et al. 2006). The transcript has an unknown function in rice but is characterized in *Arabidopsis thaliana* as “similar to heat stress transcription factor”. Although the function of CHP is unknown yet, The Rice Annotation Project Database (RAP-DB) links the transcript to the protein F2EHT9 which is further linked to the protein family of small heat-shock proteins (Hsp20) in the Uniprot database (Consortium 2015; Sakai et al. 2013). Interestingly, the expression profiles of HSFA2A and the CHP were found to be correlated (*r*_s_ = 0.943; *p* = 0.0167). Our findings might support the homology-based assumption of a linkage between the transcript Os05t0296800-02 described as CHP and an HSP20 protein. Further correlations were found between HSFA2A and the CBGs pollenless3 and OSABI5 as well as the phenotypic endpoint shoot elongation.

Increased transcript abundances of HSPs are a general response to cellular stress and damage and is also observed as an arsenite-induced effect in *A. thaliana* (Fu et al. 2014). Expression changes of transcripts coding for HSPs were also found under drought stress in rice plants. HSPs are hypothesized to be involved in cell thermotolerance as well as in tolerance to other environmental stressors such as oxidative-, chilling-, salt-, and heavy metal stresses (Gorantla et al. 2007). HSPs were also shown to regulate expression of other stress-inducible genes and were furthermore suggested to take part in the functions “metabolic process,” “DNA-dependent regulation of transcription,” and “regulation” in general, which shows the early “disturbance of homeostasis” in rice roots (Gorantla et al. 2007). This involvement in general stress is also reflected by the fact that HSFA2A was also one of the transcripts associated to the GO-terms “response to stress” and “metabolic process” which were significantly induced at both arsenite concentrations. Besides the CBGs characterized by qPCR in the current study, three additional heat-shock-related gene transcripts were identified to be differentially expressed (Os02g0527300, Os02g0758000, Os03g0266900). This finding underlines the importance of this defense mechanism in response to arsenite exposure. However, due to their weakly defined dose-dependent expression in the investigated concentration range and their function in a general stress response, the suitability of HSFA2A as a molecular biomarker for arsenite-induced stress should be assessed critically.

### Expression profiles and biological functions of UDPGT

UDPGT (Os05t0527000–01) showed distinct expression changes at 5 mg kg^−1^_dw_ added arsenite, an expression maximum at 13 mg kg^−1^_dw_ added arsenite, and a distinct expression profile with a dose-dependent expression (Fig. 2b). For the RER of UDPGT a 3-parametric sigmoidal curve fitting was calculated. As the RER of UDPGT is already high at the lowest test concentration, further analyses with lower concentrations would be necessary for the derivation of EC_x_ values. The uncomplete characterization of the UDPGT-expression at low concentrations might result in a lack of correlation between the expression profiles of UDPGT and the other CBGs as well as the phenotypic endpoints. However, for UDPGT an EC_50_ value below 5 mg arsenite kg^−1^_dw_ might be assumed. The glucosyltransferase UDPGT (UDP-glucuronosyl/UDP-glucosyltransferase family protein) is linked to the molecular functions “quercetin 3-O-glucosyltransferase activity” and “quercetin 7-O-glucosyltransferase activity”. It is thus involved in the anthocyanin pigment biosynthesis (Consortium 2015). Anthocyanins belong to the group of flavonoids that are secondary metabolites known to be associated to arsenic stress (Chakrabarty et al. 2009; Islam et al. 2015; Winkel-Shirley 2002). This might also be reflected by the induction of the GO-term “metabolic process” under both arsenite concentrations where UDPGT was one of the associated transcripts. Another identified GO-term for As_high_ exposure related to flavonoid glycoside synthesis is the “L-arabinose metabolic process” (Seigler 1999). L-arabinose is a sugar involved in flavonoid glycoside synthesis that is also related to aluminum toxicity in maize plants, possibly due to its discussed metal-binding capacity (Winkel-Shirley 2002). Flavonoids accumulate in the root tip of Arabidopsis seedlings reflecting the localization of the flavonoid biosynthetic machinery in the root elongation zone of these plants (Clemens 2006; Fu et al. 2014; Winkel-Shirley 2002).

These findings imply a possible mechanistic link between arsenite exposure and the observed inhibition of root elongation by an altered flavonoid synthesis related to expression changes of UDPGT. However, in the course of analogous DNA-microarray-experiments that were performed after exposure to sediments spiked with either cadmium, chromium (VI), chromium (III), or nickel, the root elongation was affected but UDPGT was not identified as a DEG (data not shown). Unlike this, the inhibition of root elongation and the upregulation of UDP-glucuronosyl/UDP-glucosyltransferase related transcript in rice plants was found under As(V) and Al(III) stress (Huang et al. 2009; Norton et al. 2008). Interestingly, Al(III) caused additional, similar effects on the phenotype of rice roots as observed under arsenite stress (Alvarez et al. 2012; Liu et al. 2016). Beside inhibiting root elongation, Al(III) has been described to induce a disorder of epidermal cells (Alvarez et al. 2012). This disorder resulted in less elastic primary roots, also described as root swelling, and was observed especially in Al-intolerant *indica* cultivars (Alvarez et al. 2012; Liu et al. 2016). Root swelling was also observed in the present and the previous study in the arsenite-exposed *indica* cultivar Palmar-18 (Brinke et al. 2015).

UDP-glucose was further suspected to take part in detoxification of Al(III) by masking the binding sites of Al(III) in the cell walls (Huang et al. 2009). In a recessive rice mutant with hypersensitivity to Al(III) exposed to Al(III), an upregulation of genes encoding UDP-glucuronosyl/UDP-glucosyltransferase family proteins, including also UDPGT, was observed (Huang et al. 2009). The current findings show the induction of UDPGT under As(III)-stress which might indicate related defense mechanisms for Al(III) and As(III). However, the role of UDG-glucose in the defense mechanism is reported to be linked to a bacterial-type ABC transporter with a high specificity for UDP glucose [32]. The proteins forming the named ABC-reporter are assumed to be encoded by the genes STAR1 and STAR2. These genes were described as specifically induced by aluminum. In the presented study, based on the results of the DNA-Microarray, STAR1 and STAR2 were not significantly upregulated in rice plants exposed to arsenite (Data not shown. For raw data please look up Array Express Archive of Functional Genomics Database (Experiment ArrayExpress accession: E-MTAB-5430)). Although detoxification mechanisms in rice plants are still not completely understood, it can be inferred from this similar effect on UDP-glucuronosyl/UDP-glucosyltransferase family proteins that trivalent aluminum and arsenite might induce similar molecular responses resulting in comparable phenotypic changes.

### Expression profiles and biological functions of pollenless3 and OsABI5

The expression profiles of pollenless3 (Os03t0165900–02) and OsABI5 (Os01t0859300–01) correlated significantly (*r*_s_ = 1.000; *p* = 0.00278) and also with the dose-response relationship measured for the phenotypic endpoints inhibition of root elongation (both *r*_s_ = 0.886; *p* = 0.0333) and shoot elongation (both *r*_s_ = 0.943; *p* = 0.0167). Pollenless3 is the candidate biomarker showing the highest RER = 97,28 (± 10.45) of the CBGs tested. The normalized dose-response relationships of pollenless3 and OSABI5 were fitted using a 3-parametric sigmoidal curve. The resulting EC_50_ values were 9 mg kg^−1^_dw_ for pollenless3 and 8 mg kg^−1^_dw_ for OSABI5 and thus below the EC_50_ value determined for the root elongation.

The RAP-DB links the transcript pollenless3 to the protein NP_001151272.1 that implies a similarity to a protein in *Zea mays* named pollenless3 in the NCBI database and is therefore assumed to be involved in male sterility. OsABI5 is named as “Similar to ABA response element binding factor” or “*ABA-insensitive (ABI)5* gene”, which encodes a basic leucine zipper transcription factor known to regulate processes including pathogen defense, light and stress signaling as well as seed maturation and flower development in *A. thaliana* (Jakoby et al. 2002). This is also reflected by the biological functions identified by the GO-term enrichment, namely, “pollen maturation,” “response to abscisic acid stimulus,” “response to salt stress,” “response to water deprivation,” “response to cold,” and “DNA-dependent regulation of transcription” which are significantly associated to both arsenite concentrations due to OSABI5 (Table 1). Further, a recent study reports a link between the functional term “Abscisic acid response” and arsenic stress in *A. thaliana* (Fu et al. 2014). Abscisic acid, beside other plants hormones, is also linked to the regulation of plant root elongation and development, as exogenous applied abscisic acid decreased root and shoot growth as well as lateral root formation as reviewed in Chen (2005). The relation of both genes to the GO-term “pollen maturation” suggests an impact of arsenite stress on the maturation of rice plants and supports the possible function of pollenless3. Due to the short test duration of 7 days, manifestations of possible chronic effects after arsenite exposure (e.g., sterility) were not investigated in the presented study.

In another study, the expression of OsABI5 and pollenless5 was also observed as response to arsenate in roots by cultivars Azucena and Bala that were grown hydroponically in 0 or 1 ppm arsenate and harvested after 10 days growth period (Norton et al. 2008). In a preliminary testing in frame of the presented study, rice plants of the cultivar Palmar-18 were also exposed to arsenate. Here, arsenate caused a stronger inhibition of root and shoot growth compared to arsenite. This fact was also reported by Chakrabarty et al. (2009).

### Expression profiles of CBGs on natural sediments

For a subsequent application of the identified DEGs as biomarkers for arsenite contamination of natural samples, it is essential to validate the behavior of the DEGs on natural sediments. This was done in course of the presented work by a small-scale supplementary study using natural sediments from several sources.

The natural sediments were analyzed for several metal contaminations in the < 20 μm fraction and other physicochemical parameters (see Table S1). The total arsenic concentrations of the tested sediments were in the range of the spiked concentrations in the single-substance tests on artificial sediments. To minimize interfering effects due to other sediment-contaminants than arsenic, sediments with moderate background concentration of heavy metals and low concentrations of organic chemicals were used (see Table S1) (Heininger et al. 2003). Due to differences in the TOC content of both sediments (NH-N, TOC = 4.0%_dw_ and BMAS-N, TOC = 1.4%_dw_), a normalization of the concentrations of the measured contaminants on the TOC content was conducted. In general, a higher normalized arsenic concentration in BMAS-N sediments compared to NH-N sediments was detected. These observations are in accordance with effects on the phenotype as shown by presented results. As already stated in Brinke et al. (2015), root elongation on natural sediments proved to be a less sensitive phenotypic endpoint compared to shoot elongation. Although, inhibitory effects were observed on shoot elongation, the elongation of rice roots tend to show only low inhibitory or even promotional effects. Therefore, only results from shoot elongation, as the relevant phenotypic endpoint for effects induced by contamination in natural sediments, are compared to transcriptional effects in this study. Respective results for root and shoot elongation are given in Table S10. Sediment from the locations NH-N with a total arsenic concentration of 13.3 mg kg^−1^_dw_ induced a weak stimulating effect on shoot elongation NH-N, *I* = −7.69%. Sediment from the location BMAS-N, with a total arsenic concentration of 15.6 mg kg^−1^_dw_, induced a weak inhibition of shoot elongation of BMAS-N, *I* = 8.0%. Also on the transcriptional level, sediment-induced transcriptional changes were observed. Interestingly, UDPGT was upregulated on both natural sediments [NH-N, 10.0 RER (± 8.2), BMAS-N, 6.5 RER (± 2.1)] whereas the other four CBGs were downregulated compared to the control (Table S9).

First results concerning the application of the selected CBGs showed that a response after exposure to natural sediments could be detected on the phenotypic as well on the transcriptomic level. However, a correlation between phenotypic and transcriptional effects was not observed for the natural sediments investigated in the present work. As shown by other studies, the extrapolation from artificial to natural sediments in bioassays is challenging (Höss et al. 2010) due to the complex composition of natural sediments with respect to their highly variable sediment properties and contamination patterns, as well as differences between the properties of natural and the artificial reference sediments. Besides differences in sediment contamination, (natural) sediments—as complex matrices—show fingerprint-like differences in their sediment properties such as the sediment texture or the content of organic matter that result in differences in the bioavailability of sediment-bound pollutants (Höss et al. 2010). Hence, follows that the expected upregulation of the DEGs in rice roots exposed to arsenic-contaminated sediments in the presented study might be influenced or superseded by miscellaneous parameters. First of all, even though the total arsenic concentration is in the range of concentrations that caused effects on rice-plants grown on arsenite-spiked artificial sediments, the bioavailable fraction of arsenic in natural sediments might be too low to induce changes of the transcriptome or the phenotype. An approach to address this topic would be the conduction of an exposure-dose-response experiment including the comparison between exposure concentration, internal dose (measured in root tissue) and effects on biomarker genes as well as root/shoot elongation. Such data is expected to elucidate the influence of bioavailability. This approach would be highly informative for the further characterization of arsenic stress to rice plants but because of the high amount of plant material needed for such an experiment, this approach was beyond the scope of the presented work.

Further, unlike the exposures to spiked-artificial sediments with known arsenite concentration, only the total arsenic concentration could be determined in natural sediments in the presented study. Thus, a differentiation between, for instance, the elemental speciation of inorganic arsenic compounds and the presence of organic arsenic compounds that show differences in toxicity, could not be made. Moreover, superseding effects might be caused by mixtures of the sediment contaminants and/or other physicochemical sediment properties.

To further validate the presented findings, additional natural sediments spanning a broader range of arsenic contamination are to be analyzed. The impact of arsenic speciation as well as differences in physicochemical sediment properties on the gene expression of UDPGT would be an interesting aspect for further analysis. Moreover, as phenotypical effects on shoot elongation induced by natural sediments were detected, a parallel investigation of the shoots transcriptome under arsenite stress would be an interesting approach for the future that could provide a more comprehensive picture of arsenite effects on rice plants.

## Conclusions

To sum up our results, the three CBGs UDPGT, pollenless3, and OsABI5 have shown a dose-dependent expression on arsenite-spiked artificial sediments and were therefore considered to be promising CBGs for arsenite-induced stress on artificial sediment. UDPGT even reacted more sensitively than the phenotypic endpoint. Pollenless3 and OsABI5 indicate a chronic toxicity immeasurable by means of phenotypic endpoints. A validation of the CBGs on natural sediments requires further investigation. Additionally, 48 further DEGs were identified that might be characterized concerning their suitability as CBGs for arsenite. As a further finding, we present a novel approach for the identification of biomarker genes. The combination of results from the three statistical methods for the identification of DEGs: SAM, *T* test, and Limma reduced the amount of outlier genes and identified only those genes that were strongly and stably induced and are thus potential CBGs.

## Electronic supplementary material

Further information on sediment characterization, more detailed results from qPCR and microarray analyses, information on the applied analytical methods, S1 (physiochemical parameters of sediments), S2 (RIN values of RNA samples), S3 (Primer sequences), S4 (qPCR protocols), Table S5 (Spearman Rank Order Correlation) S6 (LFCs of DEGs), S7 (Eigenvalues of principal component analyses of DEGs and whole transcriptome comparing treatments and references), S8 (CBGs and respective LFCs), S9 (mean RER of CBGs derived from exposure to arsenite-spiked artificial sediments), S10 (mean RER of CBGs derived from exposure to arsenite-contaminated natural sediments), Fig. S1 (Concentration-response curve of rice roots and shoots on arsenite-spiked sediment).

ESM 1 (PDF 761 kb)
