# Supplementary material for: Transcriptional changes measured in rice roots after exposure to arsenite-contaminated sediments
Source: Environ Sci Pollut Res Int. 2017 Nov 13;25(3):2707–17. doi: 10.1007/s11356-017-0515-z (PMC5773613; doi:10.1007/s11356-017-0515-z)
Supplement: Supplementary file 1 — (PDF 761 kb) [file 11356_2017_515_MOESM1_ESM.pdf]

**S ELECTRONIC SUPPLEMENTARY MATERIAL**

Environmental Science and Pollution Research

# TRANSCRIPTIONAL CHANGES MEASURED IN RICE ROOTS AFTER EXPOSURE TO ARSENITE-CONTAMINATED SEDIMENTS

ALEXANDRA BRINKE[a]*, GEORG REIFFERSCHEID[a], ROLAND KLEIN[b], UTE FEILER[a] and SEBASTIAN BUCHINGER[a]

[a] German Federal Institute of Hydrology, Am Mainzer Tor 1, 56068 Koblenz, Germany

[b] Department VI, Trier University, Biogeography, 54286 Trier, Germany

*Address correspondence to Alexandra.Brinke@bafg.de

Table S1 **Selected physicochemical and chemical parameters of the tested natural sediments.** The natural sediments were taken with a stainless steel van Veen grab sampler (sampling depth 0-10 cm) at two selected sampling sites (s.s.) at the German watercourse Nahe ((Niedernhausen, harbour (NH-N)), and (Bad Münster am Stein, harbour (BMAS-N))).

| | <20 µm fraction | TOC | Al | As | Cd | Cr | Cu | Li | Mn | Ni | P | Pb | Zn | Hg |
|---|---|---|---|---|---|---|---|---|---|---|---|---|---|---|
| | | % | | mg kg-$^{1}_{dw}$ | | | | | | | | | | |
| NH-N | 59,9 | 4,0 | 4,8 | 13,3 | 0,8 | 105 | 120 | 72 | 565 | 71 | 0,98 | 90 | 382 | 0,54 |
| BMAS-N | 15,6 | 1,4 | 4,5 | 15,6 | 0,7 | 64 | 53 | 68 | 1021 | 66 | 1,42 | 55 | 368 | 0,39 |

| | PCB 28 | PCB 52 | PCB 101 | PCB 118 | PCB 138 | PCB 153 | PCB 180 | PCB 31 | PCB 49 | PCB 105 | PCB 149 | PCB 156 | PCB 170 | PCB 194 | PCB 209 |
|---|---|---|---|---|---|---|---|---|---|---|---|---|---|---|---|
| | µg kg-$^{1}_{dw}$ | | | | | | | | | | | | | | |
| NH-N | 0,08 | 0,07 | 1,00 | 0,32 | 2,72 | 3,03 | 2,82 | 0,14 | 0,05 | 0,09 | 6,10 | 0,26 | 2,05 | 0,87 | 0,21 |
| BMAS-N | 0,06 | 0,05 | 2,56 | 0,53 | 7,03 | 8,07 | 8,01 | 0,10 | 0,04 | 0,08 | 15,90 | 0,65 | 4,65 | 2,26 | 0,11 |

| | a-HCH | b-HCH | g-HCH | o,p'-DDT | p,p'-DDT | o,p'-DDD | p,p'-DDD | o,p'-DDE | p,p'-DDE | 1,2,3-Trichlor-benzole | 1,3,5-Trichlor-benzole | 1,2,4-Trichlor-benzole | Penta-chlor-benzole | Hexa-chlor-benzole |
|---|---|---|---|---|---|---|---|---|---|---|---|---|---|---|
| | µg kg-$^{1}_{dw}$ | | | | | | | | | | | | | |
| NH-N | 0,08 | 0,09 | 0,03 | 0,63 | 1,81 | 2,31 | 4,83 | 0,07 | 2,86 | 0,04 | 0,05 | 1,28 | 0,06 | 1,04 |
| BMAS-N | 0,09 | 0,08 | 0,05 | 0,16 | 2,94 | 0,58 | 1,00 | 0,04 | 1,79 | 0,02 | 0,00 | 1,64 | 0,06 | 0,42 |

Table S2 **Quality of RNA** given as RIN values of RNA samples used in microarray analyses or qPCR.

| Arsenic $mg\ kg^{-1}_{dw}$ | RIN | RNA ng/µl | technique |
|---|---|---|---|
| 0 | 9,7 | 164 | microarray |
| 0 | 9,9 | 434 | microarray |
| 0 | 9,7 | 89 | microarray |
| 0 | 9,8 | 166 | microarray |
| 0 | 9.4 | 114 | microarray |
| 0 | 9.6 | 105 | microarray |
| 0 | 9.4 | 118 | microarray |
| 0 | 9.1 | 249 | microarray |
| 0 | 9.4 | 157 | microarray |
| 0 | 9.3 | 216 | microarray |
| 0 | 9.5 | 185 | microarray |
| 0 | 9.5 | 207 | microarray |
| 0 | 9.5 | 47 | microarray |
| 0 | 9.4 | 95 | microarray |
| 0 | 9.3 | 52 | microarray |
| 0 | 9.4 | 96 | microarray |
| 0 | 9.6 | 231 | microarray |
| 0 | 9.5 | 329 | microarray |
| 0 | 9.5 | 222 | microarray |
| 0 | 9.9 | 240 | microarray |
| 0 | 9.8 | 156 | microarray |
| 0 | 9.8 | 110 | microarray |
| 0 | 9.8 | 162 | microarray |
| 0 | 9.7 | 91 | microarray |
| 0 | 9.7 | 135 | microarray |
| 0 | 9.9 | 149 | microarray |
| 0 | 9.8 | 130 | microarray |
| 0 | 9.7 | 496 | microarray |
| 0 | 10 | 50 | microarray |
| 0 | 9.8 | 87 | microarray |
| 0 | 9.5 | 78 | microarray |
| 0 | 9.9 | 69 | microarray |
| 0 | 9.9 | 284 | microarray |
| 0 | 10 | 534 | microarray |
| 0 | 10 | 335 | microarray |
| 0 | 10 | 149 | microarray |
| 11 | 10 | 167 | microarray |
| 11 | 10 | 231 | microarray |
| 11 | 10 | 297 | microarray |
| 11 | 10 | 370 | microarray |
| 11 | 9.4 | 189 | microarray |
| 11 | 9.4 | 230 | microarray |
| 11 | 8.2 | 163 | microarray |
| 11 | 9.3 | 98 | microarray |
| 11 | 9.3 | 163 | microarray |

| | | | |
|---|---|---|---|
| 11 | 9.1 | 74 | microarray |
| 11 | 8.8 | 213 | microarray |
| 11 | 8.3 | 267 | microarray |
| 15 | 10 | 131 | microarray |
| 15 | 10 | 127 | microarray |
| 15 | 10 | 88 | microarray |
| 15 | 10 | 205 | microarray |
| 15 | 9.4 | 151 | microarray |
| 15 | 9 | 485 | microarray |
| 15 | 9 | 385 | microarray |
| 15 | 8 | 331 | microarray |
| 15 | 9.5 | 291 | microarray |
| 15 | 9.8 | 426 | microarray |
| 15 | 9.6 | 363 | microarray |
| 15 | 9.9 | 112 | microarray |
| 0 | 9.7 | 107 | qPCR |
| 0 | 9.2 | 181 | qPCR |
| 0 | 9.8 | 83 | qPCR |
| 5 | 9.8 | 133 | qPCR |
| 5 | 9.3 | 265 | qPCR |
| 5 | 10 | 111 | qPCR |
| 7 | 10 | 321 | qPCR |
| 7 | 9.7 | 203 | qPCR |
| 7 | 10 | 219 | qPCR |
| 9 | 10 | 304 | qPCR |
| 9 | 9.9 | 236 | qPCR |
| 9 | 10 | 240 | qPCR |
| 11 | 10 | 447 | qPCR |
| 11 | 9.5 | 141 | qPCR |
| 11 | 9.8 | 91 | qPCR |
| 13 | 10 | 436 | qPCR |
| 13 | 10 | 291 | qPCR |
| 13 | 9.7 | 109 | qPCR |
| 15 | 9.3 | 127 | qPCR |
| 15 | 10 | 210 | qPCR |
| 15 | 9.6 | 165 | qPCR |

| **Symbols** | Gene Model | Forward Primer | Reverse Primer | Length Amplicon | TM |
|---|---|---|---|---|---|
| HSFA2A | Os03t0745000-01 | 5’- gat gcg cgg ttc gtg ttt t-3’ | 5’- gac ctt gaa ccc ctg aa-3’ | 109 bp | 55°C |
| CHP | Os05t0296800-02 | 5’- gag tca tcc aaa cca cgc ca -3’ | 5’- cca aaa acc aca cgaagc tca a -3’ | 100 bp | 55°C |
| UDPGT | Os05t0527000-01 | 5’- ctt gac ggg tac gag gaa gg | 5’- tcc ctc gtc tcc acc agt tt -3’ | 119 bp | 59°C |
| OsABI5 | Os01t0859300-01 | 5’- ctc gaa gct gaa ctg aac tat ct -3’ | 5’- cct tgg act gct cca tca tt -3’ | 118 bp | 59°C |
| Pollenless3 | Os03t0165900-02 | 5’- gtg tca acg gct tca act tta t -3’ | 5’- aca cac aca cac tca caa ga -3’ | 95 bp | 59°C |
| eEF1a | AK073620 | 5’- ttt cac tct tgg tgt gaa gca | 5’- gac ttc ctt cac gat ttc atc | 103 bp | 59°C |

| | | | | | |
|---|---|---|---|---|---|
| | | gat -3’ | gta a -3’ | | |

Table S3 **Primer sequences of candidate biomarkers**

Table S4 **Primer concentrations and corresponding temperature profiles used in qPCR**

<table>
<tr><th>Primer</th><th>Primer Concentration per Reaction<br>[Forward Primer / Reverse Primer nM]</th><th>Annealing Step</th><th>Elongation Step</th></tr>
<tr><td>eEF1a</td><td>200 nM/ 200nM</td><td rowspan="2">59°C, 20 sec</td><td rowspan="2">-</td></tr>
<tr><td>HSFA2A, CHP, MS5</td><td rowspan="2">50 nM/ 50nM</td></tr>
<tr><td>UDPGT, OSABI5</td><td>55°C, 20 sec</td><td>72°C, 15 sec</td></tr>
</table>

Table S5 **Spearman Rank Order Correlation** calculated on the RER of the CBGs and the inhibition [%] of root and shoot elongation.

| | | root | HSAFA2A | CHP | UDPGT | OSBAI5 | pollenless3 |
|---|---|---|---|---|---|---|---|
| **shoot** | Spearman Rank Correlation Coefficient | 0,943 | 0,886 | 0,714 | 0,486 | 0,943 | 0,943 |
| | p-value | 0,0167* | 0,0333* | 0,136 | 0,356 | 0,0167* | 0,0167* |
| | Number of samples | 6 | 6 | 6 | 6 | 6 | 6 |
| **root** | Spearman Rank Correlation Coefficient | | 0,829 | 0,657 | 0,543 | 0,886 | 0,886 |
| | p-value | | 0,0583 | 0,175 | 0,297 | 0,0333* | 0,0333* |
| | Number of samples | | 6 | 6 | 6 | 6 | 6 |
| **HSAFA2A** | Spearman Rank Correlation Coefficient | | | 0,943 | 0,371 | 0,943 | 0,943 |
| | p-value | | | 0,0167* | 0,497 | 0,0167* | 0,0167* |
| | Number of samples | | | 6 | 6 | 6 | 6 |
| **CHP** | Spearman Rank Correlation Coefficient | | | | 0,0857 | 0,829 | 0,829 |
| | p-value | | | | 0,919 | 0,0583 | 0,0583 |
| | Number of samples | | | | 6 | 6 | 6 |
| **UDPGT** | Spearman Rank Correlation Coefficient | | | | | 0,429 | 0,429 |
| | p-value | | | | | 0,419 | 0,419 |
| | Number of samples | | | | | 6 | 6 |
| **OSBAI5** | Spearman Rank Correlation Coefficient | | | | | | 1 |
| | p-value | | | | | | 0,00278* |
| | Number of samples | | | | | | 6 |

* Significant relationship between the two variables

Table S6 **Mean expression values of the DEGs under the treatments $As_{low}$ (n=12) and $As_{high}$ (n=12) and the respective references (n=36).** The expression values are given as log2-fold changes (LFCs). Errors are given as standard error (SE) and Coefficient of variation (CV).

| | $As_{low}$ | | | | $As_{high}$ | | | |
|---|---|---|---|---|---|---|---|---|
| | Treatment | | | Reference | Treament | | | Reference |
| TargetID | LFC | SE | CV | LFC | LFC | SE | CV | LFC |
| AF513384 | 3.10 | 0.22 | 24.54 | 0.01 | 3.11 | 0.10 | 10.71 | 0.03 |
| AK070059 | 5.06 | 0.19 | 13.11 | 0.09 | 5.00 | 0.22 | 15.44 | 0.07 |
| AK121692 | 3.80 | 0.20 | 18.13 | -0.02 | 4.00 | 0.16 | 13.65 | -0.04 |
| AK242697 | 5.79 | 0.35 | 20.64 | 0.15 | 6.49 | 0.25 | 13.35 | 0.14 |
| CI189986 | 7.00 | 0.16 | 7.97 | 0.07 | 7.26 | 0.23 | 10.78 | 0.10 |
| CI261330 | 5.62 | 0.16 | 9.68 | 0.01 | 5.78 | 0.19 | 11.64 | 0.06 |
| CT834584 | -6.96 | 0.02 | -0.97 | -0.01 | -6.88 | 0.16 | -8.17 | -0.10 |
| NP13017251 | 4.08 | 0.26 | 21.73 | -0.05 | 4.37 | 0.34 | 27.31 | -0.01 |
| Os01t0241400-01 | 5.01 | 0.11 | 7.65 | 0.27 | 5.11 | 0.18 | 12.50 | 0.30 |
| Os01t0510200-01 | 4.60 | 0.15 | 11.07 | 0.04 | 4.53 | 0.12 | 9.36 | 0.02 |
| Os01t0627800-01 | 4.40 | 0.23 | 18.13 | 0.06 | 4.47 | 0.35 | 26.79 | 0.07 |
| Os01t0692000-01 | 4.27 | 0.12 | 9.98 | -0.03 | 4.23 | 0.18 | 14.37 | 0.04 |
| **Os01t0859300-01** | **5.15** | **0.20** | **13.48** | **-0.12** | **5.46** | **0.22** | **13.75** | **0.09** |
| Os02t0179600-01 | 4.80 | 0.13 | 9.34 | -0.09 | 4.82 | 0.13 | 9.49 | -0.07 |
| Os02t0259900-01 | 5.47 | 0.17 | 10.96 | -0.23 | 6.27 | 0.35 | 19.28 | -0.16 |
| Os02t0527300-01 | 4.40 | 0.20 | 16.13 | 0.12 | 4.38 | 0.14 | 11.12 | 0.12 |
| Os02t0758000-01 | 4.10 | 0.10 | 8.83 | -0.06 | 3.82 | 0.17 | 15.70 | -0.06 |
| Os03t0154000-01 | 4.99 | 0.13 | 9.06 | 0.02 | 5.07 | 0.07 | 4.71 | 0.12 |
| Os03t0165900-01 | 5.56 | 0.22 | 13.44 | 0.23 | 5.59 | 0.21 | 13.02 | 0.23 |
| **Os03t0165900-02** | **5.43** | **0.21** | **13.47** | **0.01** | **5.41** | **0.23** | **14.40** | **0.01** |
| Os03t0232800-03 | 3.87 | 0.18 | 15.95 | 0.15 | 4.09 | 0.24 | 20.37 | 0.11 |
| Os03t0266900-01 | 5.03 | 0.15 | 10.57 | -0.03 | 5.17 | 0.38 | 25.29 | -0.08 |
| Os03t0283100-02 | 3.01 | 0.13 | 14.93 | -0.04 | 3.22 | 0.17 | 18.71 | -0.02 |
| Os03t0561400-01 | 2.71 | 0.11 | 13.69 | 0.01 | 3.19 | 0.20 | 21.23 | 0.02 |
| Os03t0757600-01 | 7.94 | 0.17 | 7.24 | 0.31 | 7.85 | 0.24 | 10.50 | 0.29 |
| Os03t0820300-01 | 5.30 | 0.21 | 13.62 | 0.15 | 5.06 | 0.17 | 11.71 | 0.12 |
| Os03t0820400-01 | 3.69 | 0.16 | 14.60 | -0.01 | 3.86 | 0.21 | 18.78 | 0.11 |
| Os04t0339400-01 | 8.27 | 0.12 | 4.86 | 0.15 | 8.53 | 0.15 | 5.95 | 0.28 |
| Os04t0352400-01 | 5.10 | 0.20 | 13.44 | 0.06 | 5.28 | 0.38 | 24.90 | 0.07 |
| **Os05t0527000-01** | **7.25** | **0.27** | **12.84** | **0.10** | **7.44** | **0.31** | **14.49** | **0.10** |
| Os07t0418500-01 | 4.67 | 0.12 | 9.07 | 0.12 | 4.58 | 0.14 | 10.53 | 0.10 |

| | | | | | | | | |
|---|---|---|---|---|---|---|---|---|
| Os07t0418500-02 | 4.81 | 0.09 | 6.35 | 0.08 | 4.79 | 0.14 | 10.27 | 0.09 |
| Os07t0625400-01 | 4.86 | 0.14 | 9.84 | 0.03 | 4.90 | 0.18 | 12.56 | 0.01 |
| Os08t0174300-00 | 3.69 | 0.08 | 7.61 | -0.14 | 3.73 | 0.10 | 9.45 | -0.04 |
| Os10t0498100-01 | -4.03 | 0.13 | -11.31 | 0.10 | -4.30 | 0.20 | -16.04 | -0.18 |
| Os10t0527400-01 | 7.15 | 0.18 | 8.81 | -0.03 | 7.61 | 0.23 | 10.58 | 0.14 |
| Os10t0528300-01 | 5.29 | 0.10 | 6.29 | -0.14 | 5.60 | 0.06 | 3.77 | 0.12 |
| Os10t0568900-01 | 2.95 | 0.09 | 11.13 | -0.01 | 3.23 | 0.09 | 9.61 | 0.00 |
| Os11t0256900-01 | -6.92 | 0.13 | -6.70 | 0.02 | -7.11 | 0.20 | -9.55 | -0.18 |
| TA33989_4530 | 3.92 | 0.15 | 13.09 | 0.17 | 3.69 | 0.15 | 14.42 | 0.03 |
| TA37227_4530 | 4.68 | 0.23 | 16.70 | 0.07 | 4.59 | 0.26 | 19.79 | 0.05 |
| TA46453_4530 | 4.67 | 0.17 | 12.53 | 0.11 | 4.80 | 0.21 | 14.83 | 0.16 |
| TA47718_4530 | 5.63 | 0.12 | 7.52 | -0.12 | 6.01 | 0.06 | 3.41 | 0.08 |
| TA57069_4530 | 8.33 | 0.22 | 9.13 | 0.32 | 8.58 | 0.31 | 12.57 | 0.45 |
| **Os03t0745000-01** | **2.63** | **0.18** | **23.96** | **0.08** | **2.64** | **0.41** | **53.44** | **0.05** |
| **Os05t0296800-02** | **2.77** | **0.20** | **25.39** | **0.04** | **2.77** | **0.47** | **58.56** | **0.04** |

Table S7 **Eigenvalues of Principal Components (PC) corresponding to Figure 2.**

| | | **Description** | **PC 1** | **PC 2** |
|---|---|---|---|---|
| Arsenic 11 mg $kg^{-1}{}_{dw}$ | a | whole gene set | 28.883 % | 10.523 % |
| | b | significantly expressed genes | 85.279 % | 02.962 % |
| Arsenic 15 mg $kg^{-1}{}_{dw}$ | c | whole gene set | 27.894 % | 11.769 % |
| | d | significantly expressed genes | 90.991 % | 1.660 % |

TableS8 **Candidate biomarker for arsenite contamination** and their expression levels in microarray analysis, given as mean value of LFCs calculated of 12 treatments each. Errors are given as standard errors.

| **Symbols** | **Gene Model** | **Description (RAP-DB)** | **$As_{low}$** mean | ± | **$As_{high}$** mean | ± |
|---|---|---|---|---|---|---|
| **HSFA2A** | Os03t0745000-01 | rapdb/Similar to Heat stress transcription factor A-2a. | 5.15 | 0.20 | 5.46 | 0.22 |
| **CHP** | Os05t0296800-02 | rapd/conserved hypothetical protein. | 5.43 | 0.21 | 5.41 | 0.23 |
| **UDPGT** | Os05t0527000-01 | rapd/UDP-glucuronosyl/UDP-glucosyltransferase family protein. | 7.25 | 0.27 | 7.44 | 0.31 |
| **OsABI5** | Os01t0859300-01 | rapdb\|Similar to ABA response element binding factor (Fragment). | 2.63 | 0.18 | 2.64 | 0.41 |
| **Pollenless3** | Os03t0165900-02 | rapdb/Similar to pollenless3. | 2.77 | 0.20 | 2.77 | 0.47 |

Table S9 **Mean RER (n=9; mean ± standard error) measured in rice roots exposed to arsenite spiked artificial sediments.** Highest relative expression values are highlighted by bold text.

| | HSFA2A | | CHP | | UDPGT | | OsABI5 | | pollenless3 | |
|---|---|---|---|---|---|---|---|---|---|---|
| mg kg-1dw | r e r | ± | r e r | ± | r e r | ± | r e r | ± | r e r | ± |
| 5 | 3.52 | 2.06 | 1.70 | 0.99 | 4.88 | 1.80 | 3.21 | 1.60 | 4.35 | 1.56 |
| 7 | 2.65 | 1.30 | 1.32 | 0.05 | 6.01 | 1.06 | 4.12 | 2.03 | 11.49 | 5.16 |
| 9 | 4.09 | 0.79 | 1.49 | 0.22 | **9.10** | 2.15 | 11.38 | 3.43 | 49.41 | 24.94 |
| 11 | 4.32 | 1.34 | 2.08 | 0.25 | 7.87 | 1.33 | 15.74 | 4.13 | 81.27 | 5.26 |
| 13 | **7.07** | 1.23 | **3.79** | 1.23 | 7.37 | 0.41 | **16.61** | 4.80 | **97.24** | 10.45 |
| 15 | 4.36 | 0.75 | 3.09 | 0.86 | 7.54 | 0.63 | 16.50 | 2.58 | 85.17 | 13.87 |

Table S10 **Mean RER (n= 4; mean ± standard error) and mean inhibition of root- and shoot elongation (n=4; mean ± gaussion error propagation) measured in rice plants exposed to natural sediments.** The natural sediments were taken with a stainless steel van Veen grab sampler (sampling depth 0-10 cm) at two selected sampling sites (s.s.) at the German watercourse Nahe ((Niedernhausen, harbour (NH-N)), and (Bad Münster am Stein, harbour (BMAS-N))).

| | Arsenic | Root elongation | | Shoot elongation | | HSFA2A | | CHP | | UDPGT | | OsABI5 | | Pollenless3 | |
|---|---|---|---|---|---|---|---|---|---|---|---|---|---|---|---|
| | [mg $kg^{-1}_{dw}$] | I [%] | ± | I [%] | ± | RER | ± | RER | ± | RER | ± | RER | ± | RER | ± |
| NH-N | 13 | -7,7 | 14,7 | 8,0 | 8,6 | 0.48 | 0.37 | 0.14 | 0.04 | 10,0 | 8,2 | 0.60 | 0.31 | 0.76 | 0.46 |
| BMAS-N | 15 | -21,1 | 6,9 | -4,8 | 5,8 | 0.48 | 0.21 | 0.14 | 0.01 | 6,5 | 2,1 | 0.29 | 0.03 | 0.37 | 0.05 |

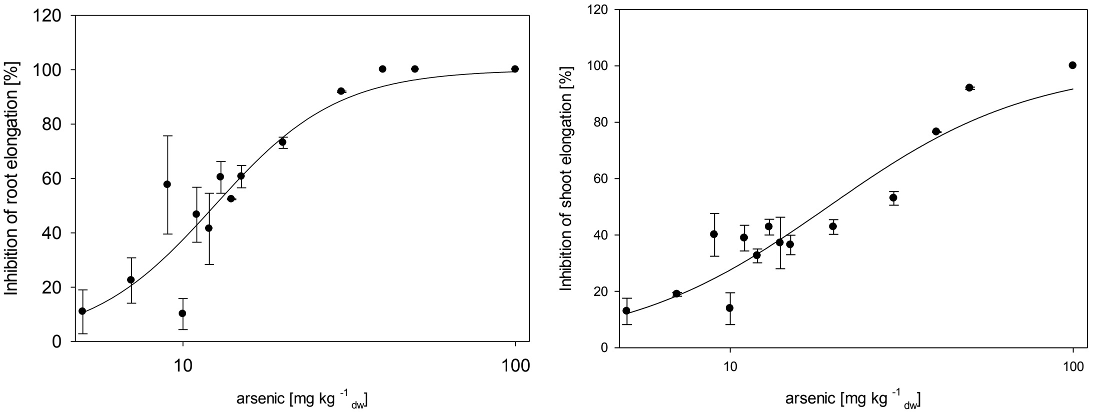


Figure S1 **Concentration-response curve for *Oryza sativa* exposed to arsenite-spiked sediments**. **expressed as a) the inhibition of root elongation with SD and b) the inhibition of shoot elongation.** The data were obtained from exposures conducted for the microarray experiments (n=3) and enhanced with results obtained in a previous study published by Brinke et al. (2015).
